# Validation of APACHE II, APACHE III and SAPS II scores in in-hospital and one year mortality prediction in a mixed intensive care unit in Poland: a cohort study

**DOI:** 10.1186/s12871-020-01203-7

**Published:** 2020-12-02

**Authors:** Szymon Czajka, Katarzyna Ziębińska, Konstanty Marczenko, Barbara Posmyk, Anna J. Szczepańska, Łukasz J. Krzych

**Affiliations:** 1 Department of Anesthesiology and Intensive Care, School of Medicine in Katowice, Medical University of Silesia, Katowice, Poland; 2 Students’ Scientific Society, Department of Anesthesiology and Intensive Care, School of Medicine in Katowice, Medical University of Silesia, Katowice, Poland

## Abstract

**Background:**

There are several scores used for in-hospital mortality prediction in critical illness. Their application in a local scenario requires validation to ensure appropriate diagnostic accuracy. Moreover, their use in assessing post-discharge mortality in intensive care unit (ICU) survivors has not been extensively studied. We aimed to validate APACHE II, APACHE III and SAPS II scores in short- and long-term mortality prediction in a mixed adult ICU in Poland. APACHE II, APACHE III and SAPS II scores, with corresponding predicted mortality ratios, were calculated for 303 consecutive patients admitted to a 10-bed ICU in 2016. Short-term (in-hospital) and long-term (12-month post-discharge) mortality was assessed.

**Results:**

Median APACHE II, APACHE III and SAPS II scores were 19 (IQR 12–24), 67 (36.5–88) and 44 (27–56) points, with corresponding in-hospital mortality ratios of 25.8% (IQR 12.1–46.0), 18.5% (IQR 3.8–41.8) and 34.8% (IQR 7.9–59.8). Observed in-hospital mortality was 35.6%. Moreover, 12-month post-discharge mortality reached 17.4%. All the scores predicted in-hospital mortality (*p* < 0.05): APACHE II (AUC = 0.78; 95%CI 0.73–0.83), APACHE III (AUC = 0.79; 95%CI 0.74–0.84) and SAPS II (AUC = 0.79; 95%CI 0.74–0.84); as well as mortality after hospital discharge (*p* < 0.05): APACHE II (AUC = 0.71; 95%CI 0.64–0.78), APACHE III (AUC = 0.72; 95%CI 0.65–0.78) and SAPS II (AUC = 0.69; 95%CI 0.62–0.76), with no statistically significant difference between the scores (*p* > 0.05). The calibration of the scores was good.

**Conclusions:**

All the scores are acceptable predictors of in-hospital mortality. In the case of post-discharge mortality, their diagnostic accuracy is lower and of borderline clinical relevance. Further studies are needed to create scores estimating the long-term prognosis of subjects successfully discharged from the ICU.

**Supplementary Information:**

The online version contains supplementary material available at 10.1186/s12871-020-01203-7.

## Background

The main goal of admitting a patient to an intensive care unit (ICU) is to reduce morbidity-related complications and, therefore, to prevent mortality due to possibly reversible severe deterioration in the clinical condition of the patient. Several simple, acknowledged tools are commonly used for outcome prediction in critical illness. These scores are based on the worst data obtained within the first 24 h post-admission and are not recalculated during the patient’s stay. Thus, the higher the scores reached, the higher the risk of in-hospital mortality. The first models estimating the risk of in-hospital death were developed over 30 years ago. The first two were the Acute Physiology and Chronic Health Evaluation (APACHE) score (1981) [1] and the Simplified Acute Physiology Score (SAPS) (1988) [2]. Since then, many attempts have been made to improve their diagnostic accuracy, and subsequent versions, including APACHE II, III, IV and SAPS II and III [3–7] were developed.

The APACHE score was created based on data from U.S. hospitals only, whereas SAPS relied on data from Europe and North America. Although all up-to-date versions of both scores have been verified in terms of their diagnostic accuracy, older scoring models (i.e. APACHE II, SAPS II) remain ‘gold standards’ in prognostication among severely ill patients in individual ICUs worldwide. Both APACHE and SAPS performance in predicting in-hospital mortality has already been verified in patients with various diagnoses [8–16]. However, their use in assessing post-discharge mortality in ICU survivors has not been studied [17–21].

One ought to remember that usability of these scores in the management of individual patients remains limited. As they were primarily developed for outcome prediction, they can be used only to compare ICU performance and quality improvement initiatives. Thus, their application in a local ICU setting requires validation to ensure appropriate diagnostic accuracy.

We therefore sought to verify the ability of three scores, i.e. APACHE II and III, and SAPS II, to predict in-hospital and post-discharge mortality in adult patients at a tertiary ICU.

## Methods

An observational prospective study was performed at a 10-bed mixed university ICU in Poland. The study covered 303 consecutive adult patients admitted between 1 January 2016 and 31 December 2016. Readmissions (*n* = 7) were excluded from the analysis. No sample size calculation was performed a priori. Under Section 21 and 22 of the Law of 5 December 1996 on the Medical Profession (Poland), due to the non-interventional design of the study, no approval of the Ethics Committee was required.

Data including demographics and comorbidities were recorded from medical records. Clinical and laboratory data were recorded on admission. Physiological data was recorded in 1-h periods. Additionally, for each patient the clinical background of admission (i.e. surgical/medical; in-hospital/ out-of-hospital) and the outcome were assessed. Data was always collected and verified independently by two researchers. Patient confidentiality was ensured as the dataset was fully anonymized. Cases with single incidents of missing data were subjected to statistical analysis.

In-hospital mortality was defined as a death occurring during the index hospitalization, regardless of the duration of the hospital stay (i.e. death occurring during an ICU stay or in another hospital ward after ICU discharge). A follow-up observation was set at 12 months. Post-discharge mortality was verified based on information acquired from the PESEL (Poland’s Universal Electronic System for Registration of the Population) database [22].

Statistical analysis was performed using MedCalc Statistical Software version 18.1 (MedCalc Software bvba, Ostend, Belgium). Continuous variables were expressed as a median and interquartile range (IQR). Qualitative variables were expressed as absolute values and/or a percentage. Between-group differences for quantitative variables were assessed using the Mann-Whitney U-test or the Kruskal-Wallis test. Their distribution was verified with the Shapiro-Wilk test. The Chi-squared test or Fisher’s exact test were applied for qualitative variables. All tests were two-tailed.

Appropriate scores of APACHE II, APACHE III and SAPS II, and their corresponding predicted mortality ratios for a whole cohort of data were calculated. Observed-to-predicted (expected) mortality rates were assessed based on the equation ‘O/P’, where ‘O’ was the number of observed in-hospital deaths and ‘P’ was the sum of individual risks of death predicted by the three scores, expressed as decimals.

Validation of APACHE II, APACHE III and SAPS II was tested by assessing discrimination and validation. Discrimination was verified using receiver operating characteristic (ROC) analysis. The ROC curves were drawn. The areas under the ROC curves (AUC) and exact binominal 95% confidence intervals (CI) for the AUCs were calculated. We used the method proposed by DeLong et al. for the assessment of the differences between AUCs. Diagnostic accuracy was defined as poor if an AUC was 0.6–0.69, acceptable if an AUC was 0.7–0.79 and excellent if an AUC was at least 0.8. Calibration was verified using calibration curves and the Hosmer-Lemeshow goodness-of-fit test, and appropriate chi-squared values were calculated. Calibration curves were drawn by plotting predicted against actual mortality for groups of the patient population stratified by 10% increments of predicted mortality (i.e. by deciles). Chi-squared values with *p* > 0.05 indicated a good fit.

A ‘*p*’ value of < 0.05 was considered statistically significant.

The TRIPOD (Transparent Reporting of a multivariable prediction model for Individual Prognosis Or Diagnosis) statement was applied in order to improve the transparency of reporting [23].

## Results

### Demography

The study group covered 160 (53%) males and 143 (47%) females. The median age of patients was 61 years (IQR 49–70). In-hospital mortality was 35.6% (i.e. 108 out of 303 patients). Moreover, 12-month post-discharge mortality reached 17.4% (i.e. 34 of 195 ICU survivors). The overall mortality was 46.9% (i.e. 142 of 303 patients). The study group characteristics are presented in Table 1.

**Table 1:** Study group characteristics, including comparison between survivors and non-survivors in the ICU observation

Variable	All patients	ICU survivors	ICU non-survivors	‘p’
	*N* = 303 (100%)	195 (64.4%)	108 (35.6%)	
Age (years)	61 [49–70]	61 [58–63]	61.5 [60–65]	0.27
Male sex	143 (47.2%)	86 (28.4%)	57 (18.8%)	0.58
Hospitalisation before ICU admission (days)	2 [1–6]	1 [0–6]	3 [1–6]	**< 0.05**
Categories of diseases				
Shock				
septic	41 (13.5%)	20 (6.6%)	21 (6.9%)	**< 0.05**
hypovolemic	15 (5%)	9 (3%)	6 (2%)	0.11
Organ failure				
Respiratory^a^	199 (65.7%)	120 (39.6%)	79 (26%)	**< 0.05**
Cardiovascular^b^	114 (37.6%)	71 (23.4%)	43 (14.2%)	0.18
acute kidney injury^c^	37 (12.2%)	13 (4.3%)	24 (7.9%)	**< 0.0001**
acute liver failure	14 (4.6%)	3 (0.9%)	11 (3.6%)	**< 0.001**
multi-organ failure	22 (7.3%)	5 (1.6%)	17 (5.6%)	**< 0.0001**
Gastrointestinal				
acute abdomen/peritonitis	34 (11.2%)	23 (7.6%)	11 (3.6%)	0.84
acute pancreatitis	7 (2.3%)	5 (1.6%)	2 (0.6%)	0.16
Other diagnosis				
severe arrythmia^d^	59 (19.5%)	29 (9.6%)	30 (9.9%)	**< 0.01**
any neurological	54 (17.8%)	47 (15.5%)	7 (2.3%)	**< 0.0001**
coma	9 (3%)	4 (1.3%)	5 (1.6%)	0.66
Admissions				
Post-operative				
planned	142 (46.9%)	116 (38.2%)	26 (8.6%)	**< 0.0001**
emergency	65 (21.5%)	39 (12.9%)	26 (8.6%)	0.07
Non-operative	96 (31,7%)	40 (13,2%)	56 (18,5%)	**< 0.0001**
Surgical				
abdominal	56 (18.5%)	47 (15.5%)	9 (3%)	**< 0.001**
neurosurgery	84 (27.7%)	64 (21.1%)	20 (6.6%)	**< 0.01**
gynecology	21 (6.9%)	16 (5.3%)	5 (1.6%)	0.24
Medical				
same hospital	83 (27.4%)	44 (14.5%)	39 (12.9%)	**< 0.05**
other hospital	21 (6.9%)	10 (3.3%)	11 (3.6%)	0.10
Scoring				
APACHE II (points)	19 [12–24]	15 [8–21]	23 [18.5–30]	**< 0.0001**
APACHE II Risk of Death (%)	25.8 [12.1–46]	18.2 [7.8–34.8]	45.6 [23.9–72.5]	**< 0.0001**
APACHE III (points)	67 [36.5–88]	52 [25–74]	86 [67.5–108]	**< 0.0001**
APACHE III Risk of Death (%)	18.5 [3.8–41.8]	9 [1.3–24.8]	40.2 [19.9–65.6]	**< 0.0001**
SAPS II (points)	44 [27–56]	37 [20–49]	55.5 [47.5–64.5]	**< 0.0001**
SAPS II Risk of Death (%)	34.8 [7.9–59.8]	19.6 [3.7–43.8]	57.5 [39.2–75.3]	**< 0.0001**
Observed mortality – N (%)	108 (35.6%)	–	–	–

Quantitative variables are expressed as median [IQR]; qualitative variables as absolute values (percent)

^a^need for mechanical ventilation

^b^systolic blood pressure < 90 mmHg for at least 1 h that is not responsive to fluid administration alone, secondary to cardiac dysfunction and associated with signs of hypoperfusion

^c^KDIGO 2012 AKI Definition doi:10.1038/kisup.2012.7

^d^atrial fibrillation, atrial flutter, supraventricular tachycardia, bradycardia, ventricular tachycardia, heart block,

### Score values and predicted mortality

The median scores of APACHE II, APACHE III and SAPS II were 19 (IQR 12–24), 67 (IQR 36.5–88), and 44 (IQR 27–56) points, respectively. The corresponding predicted in-hospital mortality ratios were 25.8% (IQR 12.1–46.0), 18.5% (IQR 3.8–41.8), and 34.8% (IQR 7.9–59.8), respectively, with observed-to-predicted mortality rates of 1.12, 1.38 and 0.96, respectively.

Table 2 presents mortality indices with regard to the source of admission. In-hospital mortality was statistically significantly higher for medical than for surgical patients. It was also higher in patients admitted from another hospital or from the emergency room (i.e. out-of-hospital admissions) compared with surgical patients transferred within our hospital from other wards (i.e. in-hospital non-medical admissions).

**Table 2:** In-hospital and post-discharge mortality by medical background and the source of the ICU admission

Type of admission		Number of patients	In-hospital mortality		Post-discharge mortality	
			Value	*p* < 0,05	Value	*p* < 0,05
In-hospital admissions	(A) Post-op surgical (abdominal)	56	9/56 (16%)	**A vs E,F,G**	9/47 (19%)	**_____**
	(B) Post-op surgical (neurosurgical)	84	20/84 (24%)	**B vs E,F,G**	5/64 (8%)	**B vs E,F,G**
	(C) Post-op surgical (gynecological)	21	5/21 (24%)	**C vs F,G**	2/16 (12%)	**_____**
	(D) Post-op surgical (all)	161	34/161 (21%)	**D vs E,F,G**	16/127 (13%)	**D vs G**
	(E) Medical (all)	83	39/83 (47%)	**E vs A,B,D**	10/44 (23%)	**E vs B**
(F) Out-of-hospital admissions (medical cases only)		38	24/38 (63%)	**F vs A,B,C,D**	4/14 (29%)	**F vs B**
(G) Transfers from another hospital (surgical & medical cases)		21	11/21 (52%)	**G vs A,B,C,D**	4/10 (40%)	**G vs B,D**

### Diagnostic performance of the studied scores

**Fig. 1:**
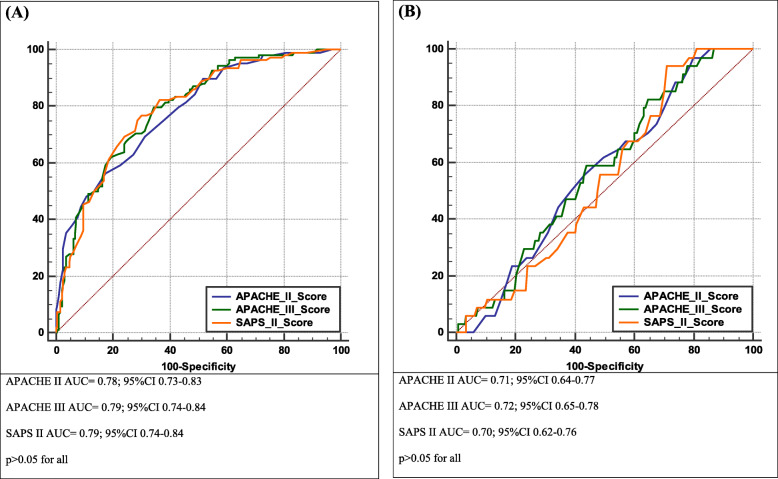
In-hospital (**a**) and post-discharge (**b**) mortality prediction by APACHE II and III, and SAPS II scores

All three investigated scores predicted in-hospital mortality with acceptable diagnostic accuracy (Fig. 1a-b). The values of AUCs for short-term mortality were ~ 0.8 whereas for long-term mortality they were ~ 0.7. The scores differed in terms of the values of AUCs in sub-analyses by type of admission (Additional file 1: Appendix 1). For in-hospital mortality, among in-hospital surgical patients the AUCs ranged from AUC = 0.81 (APACHE II) to AUC = 0.84 (SAPS II) (*p* > 0.05), whereas among in-hospital medical patients they ranged from AUC = 0.67 (APACHE II and III) to AUC = 0.71 (SAPS II) (*p* > 0.05). For post-discharge mortality, among in-hospital surgical patients the AUCs ranged from AUC = 0.67 (APACHE II) to AUC = 0.73 (APACHE III) (*p* = 0.05), whereas among in-hospital medical patients they ranged from AUC = 0.66 (APACHE III) to AUC = 0.69 (APACHE II) (*p* > 0.05).

All scores had good calibration (i.e. *p* > 0.05 for the Hosmer-Lemeshow goodness-of-fit test) (Additional file 1: Appendix 2).

## Discussion

This single-center study aimed to validate APACHE II, APACHE III and SAPS II in mortality prediction in a 10-bed ICU in Poland. We discovered that although all the scores were acceptable in predicting mortality from statistical point of view, their ability regarding 12-month prognostication proved to be limited from clinical point of view.

We found that the in-hospital ICU mortality rate was 35.6%, which was relatively high compared with international data, but lower than the value observed in the Silesia region (43.7%) [24]. The higher mortality in Polish ICUs compared with other European countries [25], which has been under debate in recent years, is rather due to differences in patient populations, indications for ICU admission, the availability of ICU beds and the organization of end-of-life care in Poland. This is also due to the skeptical attitude of some practitioners regarding guidelines on futile therapy [26, 27] and official ICU admission criteria [28]. Although patients admitted to Polish ICUs are more often at higher risk of death compared with other countries, ICU mortality observed in the Silesian Registry of Intensive Care Units was lower than that predicted by the APACHE II score [29].

In our study, APACHE II, APACHE III and SAPS II scores, and the predicted ICU mortality were as follows: 19 (IQR 12–24) points (i.e. mortality rate of 25.8%; IQR 12.1–46); 67 points (IQR 36.5–88) (mortality rate of 18.5%; IQR 3.8–41.8); and 44 points (IQR 27–56) (mortality rate of 34.8%; IQR 7.9–59.8), respectively. APACHE II and SAPS II had comparable observed-to-expected mortality ratios, close to 1.0. For APACHE III, the ratio was surprisingly high and reached 1.38. Usually, the scores overestimate mortality [30]. The cause of this phenomenon appears to be complex, and may result from substantial differences between the patient population in our unit (mixed admissions, including post-operative cases as the first priority) and the target populations these prognostic models were developed for. Medical patients were confirmed to have higher mortality than surgical patients, which is in line with previous research on this issue [31].

The reliability of the data collected is important because poor source data quality, as well as the number and type of missing physiological variables, can influence mortality predictions. In the original APACHE II study, variables were missing in 13% of cases [32]. In our data series, a total of 14% of variables were missing in all three studies’ scores which should be taken into account in data interpretation. The process of data collection is burdened with a high risk of bias. In the case of APACHE II scores, it was observed that the main causes of data errors are inconsistent choices between the highest and lowest values and problems with GCS score determination in sedated patients [32]. We used the pre-sedation GCS in sedated patients if available, data was always verified by two members of the study team independently.

Two main objective criteria are used for prognostic scales performance evaluation: namely, calibration and discrimination. Discrimination refers to the ability of a prognostic score to classify patients as survivors or non-survivors and is measured by ROC curves (i.e. AUC and 95%CI). Calibration refers to how closely the estimated probabilities of mortality correlate with the observed mortality, is of great importance for clinical trials or comparison of care between ICUs, and is depicted graphically or assessed by using goodness-to-fit models. Discrimination in our study was acceptable: all three investigated scores predicted in-hospital mortality with an AUC of almost 0.8, with no statistically significant differences between them. In terms of post-discharge mortality prediction, the diagnostic accuracy of the scores was also acceptable in terms of AUCs (i.e. > 0.7) but was rather of borderline clinical relevance (the AUC was closer to 0.5 than to 1.0, which indicates a perfectly accurate test). However, it is vital to note that the AUC itself lacks clinical interpretability as it does not reflect this. Because an AUC measures performance over all thresholds (cut-offs) for the scores, it includes both those clinically relevant and clinically illogical. Therefore, clinical interpretation of AUCs remains difficult [33].

Our observations are consistent with previous studies proving the high accuracy of the scores in short-term prognostication [31, 34–36]. Although all the scores had comparable AUCs, APACHE II and SAPS II seemed to perform better from a clinical point of view as their observed-to-expected mortality rates were 1.12 and 0.96 compared with 1.38 for APACHE III. In a study by Beck et al., who validated the same prognostic models in 16,646 adult ICU patients in the southern UK, although similarly good discrimination was reported for all three scales, calibration was imperfect [31]. The APACHE II score was more reliable than SAPS II and APACHE III in ICU patients in a study by Gilani et al. [35]. Similar findings come from a study by Khwannimit et al. who compared SAPS II and APACHE II. Although the latter model performed better in Thai ICU patients, in this case also the calibration of both scores was poor. In contrast, Sungurtekin et al. reported better prognostic accuracy for SAPS II than APACHE II in organophosphate-poisoned ICU patients [37]. Another study by Godinjak et al. demonstrated the comparable high diagnostic accuracy of APACHE II and SAPS II [36].

Calibration of our scores was good in terms of chi-squared and ‘*p*’ values. However as the application of Hosmer-Lemeshow test has been recently criticized [38], we drew the calibration curves to visualize the effect of goodness-of-fit. While the small sample size but high rate of events (i.e. deaths) is a strength of our study for the whole cohort, the calculations performed in subgroups of patients for predicted mortality were rather underpowered. On the one hand, this drawback encourages us to extend this prospective analysis to a larger group of patients. On the other hand, it must be remembered that the population of critically ill subjects changes over time and, therefore, diagnostic accuracy parameters can change dynamically [39]. Differences in the performance of scores may result from variation in the case mix, standards, the structure and organization of medical care, as well as lifestyles and genetic differences between populations [7]. Therefore, despite numerous studies performed so far on this subject, there is still a need to validate these prognostic models using data from independent samples from different ICUs in different countries, or even regions, at repeated time intervals.

Although we found some differences in the values of AUCs between surgical and medical patients, it has been confirmed by previous investigations that surgical patients generally have a better survival prognosis than medical ICU patients [6, 34]. The explanation of this fact is quite simple: in these patients the reason for ICU admission is mostly their unstable condition resulting from the performed long-lasting extensive surgical procedure, and not as much from their poor general condition prior the surgery or their comorbidities.

While all three investigated scores predicted a 12-month post-discharge mortality in a statistically significant way, their diagnostic accuracy was much lower (AUC of ~ 0.7). In a study by Angus et al. [19], the APACHE II score was also predictive of 1-year mortality (AUC of 0.671) in patients undergoing liver transplants. In contrast, a study by Lee et al. reported no relation between the scores calculated on admission and post-discharge mortality [40]. Lower diagnostic accuracy in predicting long-term mortality could be due to various reasons. The scores are calculated during the first 24 h following admission, using the worst results. The treatment implemented during ICU stay, eventual complications and the quality of the follow-up care and rehabilitation, influence the patient’s outcome and can change the results provided by the scoring systems. Lee et al. found that the discharge APACHE II score was a good predictor of post-ICU mortality and readmission [40]. Therefore, it would be more reasonable to focus on the scores calculated to estimate the long-term prediction of the patients on their discharge from the ICU. Because currently available tools have not been initially designed for such an application, further studies should be conducted to create scores estimating the long-term prediction. In this context, one ought to bear in mind that proper screening and accurate identification of patients who will stay at risk after their successful discharge from the ICU may be of great importance in order to avoid ICU readmissions, further deterioration of quality of life and higher post-discharge mortality.

The present study has some limitations. Those related to validation have been described above. However, one ought to remember also that as a single-center study, there may be bias with regard to the heterogeneous population and relatively small sample size. The final results in the scores may be affected by the confounding effect of the data selection process and the calculation of Glasgow Coma Scale results. The follow-up period in our study was limited to 12 months after the date of ICU admission. Finally, we did not include the SOFA score into our analysis. However, as this particular scoring system was primarily created for prognostication among septic patients, it seems less comprehensive in the mixed ICU setting than APACHE or SAPS [41].

## Conclusions

All the scores are acceptable predictors of in-hospital mortality. In the case of post-discharge mortality, their diagnostic accuracy is lower and of borderline clinical relevance. Further studies are needed to create scores estimating the long-term prognosis of patients successfully discharged from the ICU.

## Supplementary Information

**Additional file 1:** **Appendix 1.** Mortality prediction by APACHE II, APACHE III and SAPS II by type of admission. **Appendix 2.** Calibration plots for APACHE II, APACHE III and SAPS II for short-term (A) and long-term (B) mortality.

## Data Availability

The datasets used and analysed during the current study are available from the corresponding author on reasonable request.

## Abbreviations

ICU: Intensive care unit
APACHE: Acute Physiology and Chronic Health
SAPS: Simplified Acute Physiology Score
PESEL: Poland’s Universal Electronic System for Registration of the Population
IQR: Interquartile range
ROC: Receiver operating characteristic
AUC: Areas under (ROC) curves
TRIPOD: Transparent Reporting of a multivariable prediction model for Individual Prognosis Or Diagnosis
GCS: Glasgow Coma Scale
SOFA: Sequential Organ Failure Assessment
