# Supplementary material for: Validation of APACHE II, APACHE III and SAPS II scores in in-hospital and one year mortality prediction in a mixed intensive care unit in Poland: a cohort study
Source: BMC Anesthesiol. 2020 Dec 2;20:296. doi: 10.1186/s12871-020-01203-7 (PMC7709291; doi:10.1186/s12871-020-01203-7)
Supplement: Supplementary file 1 — Additional file 1: Appendix 1. Mortality prediction by APACHE II, APACHE III and SAPS II by type of admission. Appendix 2. Calibration plots for APACHE II, APACHE III and SAPS II for short-term (A) and long-term (B) mortality. [file 12871_2020_1203_MOESM1_ESM.docx]

APPENDIX 1. Mortality prediction by APACHE II, APACHE III and SAPS II by type of admission

| Type of admission | | In-hospital mortality | Post-discharge mortality |
| --- | --- | --- | --- |
| In-hospital admissions | Post-op surgical (abdominal) | APACHE II AUC=0.84; 95%CI 0.72-0.93  APACHE III AUC=0.86; 95%CI 0.74-0.94  SAPS II AUC= 0.79; 95%CI 0.66-0.89  p>0.05 for all | APACHE II AUC=0.58; 95%CI 0.42-0.72  APACHE III AUC=0.64; 95%CI 0.48-0.77  SAPS II AUC=0.58; 95%CI 0.43-0.72  p>0.05 for all |
|  | Post-op surgical (neurosurgical) | APACHE II AUC=0.80; 95%CI 0.70-0.88  APACHE III AUC= 0.84; 95%CI 0.74-0.91  SAPS II AUC= 0.86; 95%CI 0.77- 0.93  p>0.05 for all | APACHE II AUC= 0.64; 95%CI 0.51-0.76  APACHE III AUC= 0.70; 95%CI 0.57-0.80  SAPS II AUC= 0.72; 95%CI 0.59-0.82  APACHE II vs. SAPS II p=0.02 |
|  | Post-op surgical (gynecological) | APACHE II AUC= 0.94; 95%CI 0.74-0.99  APACHE III AUC= 0.95; 95%CI 0.76-0.99  SAPS II AUC= 0.94; 95%CI 0.74-0.99  p>0.05 for all | APACHE II AUC= 0.89; 95%CI 0.64-0.99  APACHE III AUC= 0.89; 95%CI 0.64-0.99  SAPS II AUC= 0.96; 95%CI 0.74-1.00  p>0.05 for all |
|  | Post-op surgical (all) | APACHE II AUC= 0.81; 95%CI 0.74-0.87  APACHE III AUC= 0.84; 95%CI 0.78-0.90  SAPS II AUC= 0.84; 95%CI 0.77-0.89  p>0.05 for all | APACHE II AUC= 0.67; 95%CI 0.58-0.75  APACHE III AUC= 0.73; 95%CI 0.64-0.80  SAPS II AUC= 0.70; 95%CI 0.62-0.78  APACHE II vs. APACHE III p=0.05 |
|  | Medical (all) | APACHE II AUC= 0.67; 95%CI 0.56-0.77  APACHE III AUC= 0.67; 95%CI 0.56-0.77  SAPS II AUC= 0.71; 95%CI 0.60-0.80  p>0.05 for all | APACHE II AUC= 0.69; 95%CI 0.53-0.82  APACHE III AUC= 0.66; 95%CI 0.50-0.79  SAPS II AUC= 0.67; 95%CI 0.51-0.80  p>0.05 for all |
| Out-of-hospital admissions (medical cases only) | | APACHE II AUC= 0.70; 95%CI 0.53-0.84  APACHE III AUC= 0.74; 95%CI 0.57-0.86  SAPS II AUC= 0.74; 95%CI 0.57-0.87  p>0.05 for all | APACHE II AUC= 0.89; 95%CI 0.61-0.99  APACHE III AUC= 0.89; 95%CI 0.61-0.99  SAPS II AUC= 0.60; 95%CI 0.31-0.84  p>0.05 for all |
| Transfers from another hospital (surgical & medical cases) | | APACHE II AUC= 0.82; 95%CI 0.60-0.95  APACHE III AUC= 0.83; 95%CI 0.61-0.96  SAPS II AUC= 0.79; 95%CI 0.56-0.94  p>0.05 for all | APACHE II AUC= 0.73; 95%CI 0.37-0.95  APACHE III AUC= 0.71; 95%CI 0.36-0.94  SAPS II AUC= 0.69; 95%CI 0.34-0.93  p>0.05 for all |

APPENDIX 2. Calibration plots for APACHE II, APACHE III and SAPS II for short-term (A) and long-term (B) mortality

A. short-term

Hosmer-Lemeshow goodness-of-fit, chi-squared value=13.74, test p=0.09

Hosmer-Lemeshow goodness-of-fit test, chi-squared value=6.51, p=0.59

Hosmer-Lemeshow goodness-of-fit test, chi-squared value=9.11, p=0.33

(B) long-term mortality

Hosmer-Lemeshow goodness-of-fit test, chi-squared value=6.15, p=0.63

Hosmer-Lemeshow goodness-of-fit test, chi-squared value=5.57, p=0.59

Hosmer-Lemeshow goodness-of-fit test, chi-squared value=12.1, p=0.09
